# Leadless Pacemaker Implantation in the Emergency Bradyarrhythmia Setting: Results from a Multicenter European Registry

**DOI:** 10.3390/medicina59010067

**Published:** 2022-12-28

**Authors:** Marco Schiavone, Annalisa Filtz, Alessio Gasperetti, Alexander Breitenstein, Pietro Palmisano, Gianfranco Mitacchione, Simone Gulletta, Gian Battista Chierchia, Elisabetta Montemerlo, Giovanni Statuto, Giulia Russo, Michela Casella, Francesco Vitali, Patrizio Mazzone, Daniel Hofer, Gianmarco Arabia, Fabrizio Tundo, Diego Ruggiero, Nicolai Fierro, Massimo Moltrasio, Matteo Bertini, Antonio Dello Russo, Ennio C. L. Pisanò, Paolo Della Bella, Giovanni Rovaris, Carlo de Asmundis, Mauro Biffi, Antonio Curnis, Claudio Tondo, Ardan M. Saguner, Giovanni B. Forleo

**Affiliations:** 1 Cardiology Unit, Luigi Sacco University Hospital, 20131 Milan, Italy; 2 Department of Systems Medicine, University of Rome Tor Vergata, 00133 Rome, Italy; 3 Department of Cardiology, Johns Hopkins University, Baltimore, MD 21218, USA; 4 Department of Clinical Electrophysiology and Cardiac Pacing, Centro Cardiologico Monzino, IRCSS, 20138 Milan, Italy; 5 Cardiology Department, University Hospital Zurich, 8091 Zurich, Switzerland; 6 Cardiology Unit, “Card. G. Panico” Hospital, 73039 Tricase, Italy; 7 Department of Cardiology, Spedali Civili Hospital, University of Brescia, 25121 Brescia, Italy; 8 Arrhythmology and Electrophysiology Unit, San Raffaele Hospital, IRCCS, 20132 Milan, Italy; 9 Heart Rhythm Management Centre, Universitair Ziekenhuis Brussel, Postgraduate Program in Cardiac Electrophysiology and Pacing, European Reference Networks Guard-Heart, Vrije Universiteit Brussel, 1090 Brussels, Belgium; 10 Department of Cardiology, ASST Monza, San Gerardo Hospital, 20900 Monza, Italy; 11 Department of Cardiology, IRCCS, Azienda Ospedaliero Universitaria Di Bologna, Policlinico Di S.Orsola, 40138 Bologna, Italy; 12 U.O.S.V.D. Elettrofisiologia Cardiologica—Ospedale “V. Fazzi”, 73100 Lecce, Italy; 13 Cardiology and Arrhythmology Clinic, University Hospital “Umberto I-Salesi-Lancisi”, 60123 Ancona, Italy; 14 Cardiology Unit, Sant’Anna University Hospital, University of Ferrara, 44121 Ferrara, Italy; 15 Department of Biomedical, Surgical and Dental Sciences, University of Milan, 20122 Milan, Italy

**Keywords:** leadless pacemaker, emergency bradyarrhythmia, complete AV block, slow atrial fibrillation

## Abstract

*Background.* Data on leadless pacemaker (LPM) implantation in an emergency setting are currently lacking. *Objective.* We aimed to investigate the feasibility of LPM implantation for emergency bradyarrhythmia, in patients referred for urgent PM implantation, in a large, multicenter, real-world cohort of LPM recipients. *Methods.* Two cohorts of LPM patients, stratified according to the LPM implantation scenario (patients admitted from the emergency department (ED+) vs. elective patients (ED−)) were retrieved from the iLEAPER registry. The primary outcome of the study was a comparison of the peri-procedural complications between the groups. The rates of peri-procedural characteristics (overall procedural and fluoroscopic duration) were deemed secondary outcomes. *Results.* A total of 1154 patients were enrolled in this project, with patients implanted due to an urgent bradyarrhythmia (ED+) representing 6.2% of the entire cohort. Slow atrial fibrillation and complete + advanced atrioventricular blocks were more frequent in the ED+ cohort (76.3% for ED+ vs. 49.7% for ED−, *p* = 0.025; 37.5% vs. 27.3%, *p* = 0.027, respectively). The overall procedural times were longer in the ED+ cohort (60 (45–80) mins vs. 50 (40–65) mins, *p* < 0.001), showing higher rates of temporary pacing (94.4% for ED+ vs. 28.9% for ED−, *p* < 0.001). Emergency LPM implantation was not correlated with an increase in the rate of major complications compared to the control group (6.9% ED+ vs. 4.2% ED−, *p* = 0.244). *Conclusion.* LPM implantation is a feasible procedure for the treatment of severe bradyarrhythmia in an urgent setting. Urgent LPM implantation was not correlated with an increase in the rate of major complications compared to the control group, but it was associated with longer procedural times.

## 1. Introduction

During the last decade, leadless pacemakers (LPMs) have turned into a well-established alternative to traditional transvenous (TV) pacemakers (PMs) for bradyarrhythmia treatment, especially in cases of high infective risk or vascular access concerns [1,2]. The increasing use of LPMs is due to the lower risk of lead and pocket-related complications that have been traditionally associated with TV-PMs [3]. When compared to TV-PMs, LPMs have been demonstrated to reach a reduction of 51% of major complications in the early post-procedural period (within 6 months), with up to 48–63% at one year [4,5]. Further recent evidence has shown that LPMs have an adequate electrical performance during follow-up, with a low incidence of adverse events as well (1.77% at 1 year) [1]. These advantages are of pivotal importance, especially in young patients, potentially facing several years of cardiac pacing [6]. Although recent evidence has shown that younger patients tend to have a slightly higher pacing threshold at the mid-term follow-up, the device-related complication rate is similar to what is expected in the traditional LPM patient cohort [7]. However, first-generation LPMs were able to pace only the ventricular chamber, limiting their use to atrial fibrillation (AF) with a slow ventricular rate setting and to patients who have absolute or relative contraindications for traditional TV-PMs [8]. The recent introduction of second-generation LPMs, capable of VDD pacing, may help reach AV synchronous pacing [9,10], providing an interesting alternative for patients with complete atrioventricular (AV) blocks. Although several studies have confirmed these data in an elective setting so far, very scarce data have been provided in an emergency setting. Only a small observational study from Marschall et al. [11] provided data on the safety and feasibility of the LPM implantation in an urgent setting. Therefore, we aimed to investigate the feasibility of LPM implantation for emergency bradyarrhythmias in patients referred for urgent PM implantation in a large, multicenter, real-world cohort of LPM recipients.

## 2. Methods

### 2.1. Registry Population

The i-LEAPER project (International Leadless Pacemaker Registry) is a European, multi-center, open-label, independent, and physician-initiated observational registry. A total of 12 public and private healthcare institutions from 3 different countries in Europe (Italy, Switzerland, and Belgium) were involved in this project. All consecutive patients undergoing the implantation of an LP device currently approved in Europe for clinical use (Micra™ MC1VR01 or Micra™ AVMC1AVR1 Transcatheter Pacing System—Medtronic, Inc., Minneapolis, MN, USA) from June 2015 to December 2021 were enrolled in the current registry and were used for the current analysis. This manuscript has been drafted in accordance with the tenets of the Helsinki Declaration and has been approved by the local institutional review board.

### 2.2. Data Collection, Cohort, and Outcome Definition

The demographics, patient medical history, and peri-procedural data were extracted and collected into a centralized de-identified spreadsheet, clearly defining each research item.

Patients were classified into two cohorts depending on the device implantation setting:

- Patients referred from the emergency department (ED) for LPM implantation due to urgent bradyarrhythmias (ED+);
- Patients referred for LPM implantation in a non-urgent scenario, with the procedure performed in an elective setting (ED−).

The primary outcome of the study was the comparison of the peri-procedural complications between the two cohorts. The rates of peri-procedural characteristics, seen as the overall procedural and fluoroscopic duration, were deemed secondary outcomes.

### 2.3. Statistical Analysis

Continuous variables were reported as the mean ± standard deviation (s.d.) or as the median (inter-quartile range (1st–3rd quartile) (IQR)) if normally or non-normally distributed, respectively. Categorical variables were reported as a count (%). Comparisons were performed using an Χ^2^ test or a Fisher’s exact test between categorical variables, and a Student’s *t*-test or a Mann–Whitney U test between numerical variables, as appropriate according to their distribution. Associations between predictors and outcomes of interest were tested using univariate and multivariate regression models. A parsimonious model including only variables reaching a *p* < 0.10 in a univariate analysis was built to adjust for confounders. All two-tailed *p* values < 0.05 were considered significant. All analyses were performed using STATA 14.0 (StataCorp LLC, College Station, TX, USA).

## 3. Results

### 3.1. Baseline Characteristics of the Study Cohort

A total of 1154 patients were enrolled in the current study. Patients who were implanted with an LPM due to emergency bradyarrhythmia represented 6.2% of the entire cohort (*n* = 72). The baseline characteristics of the study cohort, evaluating the differences between the two groups, are reported in Table 1. Regarding gender, no significant differences between the two groups were detected, with 33.3% of the ED+ cohort and 35.9% of the ED− cohort being male (*p* = 0.812). The two study groups were also similar regarding age characteristics (78.9 ± 15.5 years for ED+ vs. 77.0 ± 12.8 years for ED−, *p* = 0.330), body mass index (BMI) (24.3 (22.0–27.5) for ED+ vs. 25.8 (23.1–28.6) for ED−, *p* = 0.893), and cardiovascular risk factors (diabetes: ED+ cohort, 25.0% vs. ED− cohort, 22.1%, *p* = 0.672; hypertension: ED+ cohort, 54.2% vs. ED− cohort, 53.4%, *p* = 0.998). The two cohorts were balanced in terms of baseline structural heart diseases (HD), with similar rates of coronary artery disease (22.2% for ED+ vs. 23.1% for ED−, *p* = 0.996) and congenital HD (1.4% for ED+ vs. 0.8% for ED−, *p* = 0.478), apart from different rates of valvular HD (38.9% for ED+ vs. 23.7% for ED−, *p* = 0.037) and heart failure (HF) (20.8% for ED+ vs. 9.8% for ED−, *p* = 0.020). No differences were detected between the groups regarding previous rates of overall cardiac surgical procedures (18.1% for ED+ vs. 14.1% for ED−, *p* = 0.401) and coronary artery bypass grafting (CABG) (5.5% for ED+ vs. 6.4% for ED−, *p* = 0.995). Similar rates of chronic kidney disease (5.5% for ED+ vs. 4.1% for ED−, *p* = 0.539) were found in the two groups. Lastly, regarding drugs with a potential impact on PM implantation outcomes, the study cohorts showed an overall similar rate of oral anticoagulants use (87.5% for ED+ vs. 86.1% for ED−, *p* = 0.929).

### 3.2. LP Indications and Peri-Procedural Characteristics

The main indications for implanting an LPM are reported in Table 2. A total of 102 Micra™-AV devices (8.8%) were implanted in the overall cohort, with no significant differences between the groups (13.8% for ED+ vs. 8.5% for ED−, *p* = 0.204). In the ED+ cohort patients, the most common bradyarrhythmia leading to an LPM implantation was slow atrial fibrillation (AF) (76.3% for ED+ vs. 49.7% for ED−, *p* = 0.025), while in the elective setting, the sinus node arrest rates (4.2% for ED+ vs. 16.4% for ED−, *p* = 0.008) were higher. Complete and advanced atrioventricular (AV) blocks were more frequent in the ED+ cohort (37.5% vs. 27.3%, *p* = 0.027). No differences in the underlying rate of cardioinhibitory syncope was found between the groups.

Peri-procedural characteristics are reported in Table 3. Similar rates of LPM implantations via right femoral access (95.8% for ED+ vs. 96.0% for ED−, *p* = 1.000) and femoral vein angiography (22.2% for ED+ vs. 33.1% for ED−; *p* = 0.200) were found between the groups. On the other hand, different rates of temporary pacing use were found between the groups: 94.4% for ED+ vs. 28.9% for ED−, *p* < 0.001. As for delivery attempts (1 (1–1) for ED+ vs. 1 (1–1) for ED−, *p* = 1.000), rates of device repositioning (8.3% for ED+ vs. 12.3% for ED−, *p* = 0.068), and the number of patients with > 1 delivery attempts (23.6% for ED+ vs. 13.8% for ED−, *p* = 0.885), no statistical differences were found between the groups. If the rates of septal (90.3% for ED+ vs. 66.4% for ED−, *p* = 0.086) and right ventricular outflow tract (RVOT) positioning did not show statically significant differences between the groups, apical positioning was more frequent in the elective setting (9.7% for ED+ vs. 31.4% for ED−, *p* < 0.001).

The overall procedural times were longer in the ED+ cohort (60 (45–80) mins for ED+ vs. 50 (40–65) mins for ED−, *p* < 0.001), but no differences were found regarding fluoroscopy times (6.5 (5.0–9.7) mins for ED+ vs. 5.1 (3.1–9) mins for ED−, *p* = 0.103). Specifically, the time spent for positioning temporary pacing catheters was accounted for in the overall procedural times, whenever they were positioned in the electrophysiology lab, simultaneously (prior) to device implantation. The relationship between LPM implantation procedural times and baseline characteristics were tested through univariate and multivariate regression models (Table 4). In the univariate analysis, the age (OR 0.060, CI (−0.222–0.016); *p* = 0.091), BMI (OR 0.263, CI (0.041–0.955), *p* = 0.072), and ED+ setting (OR 3.451, CI (6.173–19.717); *p* < 0.001) was significantly associated with longer procedural times; in the multivariate analysis, only implanting the LPM due to urgent bradyarrhythmia (ED+ setting) remained associated with the outcome of interest (OR 5.156, CI (4.610–24.872), *p* = 0.004). The post-procedural electrical performance of the LPM was similar in the two groups in terms of sensing (10 (8–12.8) for ED+ vs. 10 (7.5–13.5) for ED−mV, *p* = 0.985), impedance (695 (570–797) ohm for ED+ vs. 690 (580–810) ohm for ED−, *p* = 0.476), and the threshold (0.50 (0.38–0.99) V for ED+ vs. 0.42 (0.28–0.70) V for ED−, *p* = 0.428), while the in-hospital stay was significantly longer in the ED+ setting (7 (3–16) days for ED+ vs. 3 (2–5) days for ED−, *p* < 0.001). Among a total number of 50 peri-procedural complications, no differences were detected in the two groups in terms of the overall rate (6.9% for ED+ vs. 4.2% for ED−, *p* = 0.244) and the single outcomes of interest (pericardial effusion and tamponade, femoral vascular injury, device dislodgement or embolization, and groin hematoma).

## 4. Discussion

This study represents the first focused analysis of LPM implantation used as a treatment for emergency bradyarrhythmias. In this insight from a large real-world LPM registry, we explored the feasibility and the complication rate of urgent LPM implantation, as compared with elective LPM treatment.

The main findings of our study can be summarized as follows:

(1) LPM implantation is a feasible procedure for the treatment of severe bradyarrhythmias in an urgent setting, in patients admitted from the ED;
(2) Emergency LPM implantation was not correlated with an increase in the rate of major complications compared to the control group (6.9% for ED+ vs. 4.2% for ED−, *p* = 0.244);
(3) LPM implantation for severe bradyarrhythmia is associated with longer procedural times (60 (45–80) mins vs. 50 (40–65) mins, *p* < 0.001), even when controlling for confounders (OR 5.156, CI (4.610–24.872), *p* = 0.004).

### 4.1. LPM for Emergency Bradyarrhythmia Treatment: Technical Aspects

Traditional transvenous pacemakers (TV-PMs) are a well-established therapy for the treatment of bradyarrhythmia, even in acute and emergency settings [12]. In the last years, LPMs have emerged as a new form of permanent pacing therapy [13,14,15], mainly for patients with slow AF [16,17]. However, the recent introduction of a more advanced pacing mode, providing AV synchronous pacing (VDD pacing), has extended its indication to patients with AV blocks with preserved sinus node function requiring permanent pacing [18], provided that an adequate follow-up is established [19]. As for the patients’ characteristics, the main indications for LPM implantation include obstruction of the venous route used for standard PM implantation, pocket issues, or a high risk of infection (Class IIa, B) [20]. Moreover, LPMs may be considered as an alternative to standard single-lead ventricular pacing, taking into consideration life expectancy (Class IIb, C) [20]. After approval studies [8,9,16,17,21] have analyzed the LPM implantation feasibility and outcomes, LPMs have shown an optimal safety and efficacy profile in terms of low and stable pacing thresholds (PTs), with a consistent performance in real-life settings as well [4,8,16,22,23]. In addition, compared to TV-PMs, LPMs have been shown to reduce complication rates and to be a feasible therapeutic alternative for selected groups of patients [24,25]. However, few data have been provided on the feasibility of LPM implantation in an emergency setting, which still represents an extremely common scenario in the current clinical practice. Indeed, only a small observational study from Marschall et al. [11] provided data on 25 elderly patients implanted with an LPM in this setting. In this study, these patients were compared to a control group of standard PM care (*n* = 53). The authors concluded that the LPM was a feasible alternative to conventional TV-PM, provided that the patients undergoing implantation were carefully evaluated and selected by the managing physicians, despite their older age.

In our study, for the first time, we evaluated the outcomes and peri-procedural characteristics of LPM implantations by comparing patients that had an LPM implanted for an emergency bradyarrhythmia requiring urgent PM therapy with patients receiving the LPM in a more common elective scenario. In our cohort, for the patients receiving the LPM in urgent scenario (ED+ cohort), the two main indications were, as expected, a slow AF and advanced and complete AV blocks, known to be the most common bradyarrhythmia that potentially requires urgent PM implantation. The implant procedure was characterized by a statistically significant difference in temporary pacing compared to the control group. This was possibly related to the more frequent use of temporary pacing, requiring another venous access as well, which is almost mandatory in such emergency cases. The overall procedural duration was acceptable at 60 (45–80) mins, even if higher than the time reported by Marschall et al. [11], showing an overall procedural duration of 39.9 ± 8.7 mins. Comparisons between the two cohorts are difficult due to the lack of other clinical data reported by these authors, such as the rate of previous cardiac surgical procedures or other underlying associated diseases, which are known to complicate these procedures from a technical point of view. As for other technical aspects, no significant differences in the overall fluoroscopy times were found between the two groups of our study, but there was a mild trend towards significance regarding longer fluoroscopy times in the ED+ group, with 6.5 (5.0–9.7) mins, vs. the ED− group, with 5.1 (3.1–9) mins (*p* = 0.103). Indeed, despite the extensive number of patients included in this study, due to the relatively modest number of patients included in the ED+ group, this study could be underpowered to detect significant differences regarding fluoroscopy times. Thus, overall longer fluoroscopy times in the ED+ group, although not statistically significant, might have contributed to increased overall procedural times in the ED+ group.

### 4.2. LPM for Emergency Bradyarrhythmia Treatment: Peri-Procedural Outcomes

Regarding the overall peri-procedural major complication rate, no significant differences were found between the two groups (ED+, 6.9% vs. ED−, 4.2%; *p* = 0.244). Previous studies have reported overall comparable complication rates in LPM implantation (3.3–7.6%), even in the elderly population, which is characterized by greater frailty and a greater risk of complications [8,26,27]. When compared to the only other study that has evaluated peri-procedural LPM-related complications in this setting, Marschall et al. [11] reported *n* = 3 deaths (12% of the entire cohort) and *n* = 0 complications; this latter small sample does not allow us to make a proper comparison between the cohorts. However, the same rate of complications found between the two study groups of our cohort corroborates the safety profile of LPM implantations, even as an emergency procedure. Moreover, when evaluating the electrical parameters after LPM implantation, no significant differences were found between the groups, supporting the feasibility and the overall acceptable results of LPM implantations in this scenario, which was shown by Marschall et al. [11] as well.

Furthermore, although the procedure was performed in an emergency setting, the proper septal targeting of the LPM deployment, known to be associated with a narrower paced QRS [28], was not undervalued, even with a tendency towards significance when compared to the elective setting (septal position, ED+, 90.3% vs. ED−, 66.4%, *p* = 0.080). Lastly, the longer in-hospital stay documented in the ED+ cohort could be partially explained by the slighter, but more severe, baseline characteristics of the ED+ cohort, showing higher rates of HF (ED+, 20.8% vs. ED−, 9.8%; *p* = 0.037) and valvular HD (ED+, 38.9% vs. ED−, 23.7%, *p* = 0.020). One may also speculate that patients admitted from the ED would undergo further clinical tests more frequently than elective patients during hospitalization, but the systematical collection of these data was lacking in our cohort.

### 4.3. Limitations

A limitation of this study is the retrospective, non-randomized nature of our multicentered registry. Despite the fact that this represents the largest cohort of patients undergoing LPM implantation in an emergency scenario, the study might have been underpowered to detect significant differences in the complication rates due to the overall low number of events detected in our registry. Moreover, many of the centers involved in this project were third-level referral centers in their region; therefore, it is not certain that similar results with comparable low complication rates could be achieved by less experienced operators. Nevertheless, we believe that despite these limitations, our study reflects a real-life experience demonstrating that LPMs may be a feasible option in an urgent setting, provided that a careful evaluation of the patients’ characteristics is performed, and the procedure is supported by experienced teams.

### 4.4. Conclusions

LPMs represent a significant technological breakthrough in the field of cardiac pacing, being feasible even in patients admitted to the ED for emergency bradyarrhythmias. Whenever appropriate for the underlying arrhythmias and the patient’s baseline clinical characteristics, our results show that LPMs may represent a valuable alternative to TV-PMs whenever implanted in referral centers by experienced and qualified operators, even as an urgent treatment.

## Figures and Tables

**Table 1:** Baseline characteristics of the study cohort.

	ED+ Cohort (*n* = 72)	ED− Cohort (*n* = 1082)	*p*
Age (years), mean ± st. dev.	78.9 ± 15.5	77.0 ± 12.8	0.330
Male, *n* (%)	24 (33.3)	389 (35.9)	0.812
BMI, median (IQR)	24.3 (22.0–27.5)	25.8 (23.1–28.6)	0.893
Diabetes, *n* (%)	18 (25.0)	239 (22.1)	0.672
Hypertension, *n* (%)	39 (54.2)	582 (53.4)	0.998
CAD, *n* (%)	16 (22.2)	250 (23.1)	0.996
Previous cardiac surgery, *n* (%)	13 (18.1)	153 (14.1)	0.401
CABG, *n* (%)	4 (5.5)	69 (6.4)	0.995
Congenital HD, *n* (%)	1 (1.4)	9 (0.8)	0.478
Valvular HD, *n* (%)	28 (38.9)	257 (23.7)	**0.037**
CKD needing hemodialysis, *n* (%)	4 (5.5)	44 (4.1)	0.539
HF, *n* (%)	15 (20.8)	106 (9.8)	**0.020**
LVEF (%), mean ± st. dev.	55 (47–61)	56 (52–61)	0.052
OAC, *n* (%)	63 (87.5)	932 (86.1)	0.929

**Abbreviations:** AF = atrial fibrillation; AFl = atrial flutter; BMI = body mass index; CABG = coronary artery bypass grafting; CAD = coronary artery disease; CIED = cardiac implantable electronic device; CKD = chronic kidney disease; HD = heart disease; HF = heart failure; LVEF = left ventricular ejection fraction; OAC = direct oral anticoagulants. Bold indicates statically significant values.

**Table 2:** LPM implantation indications.

	ED+ Cohort (*n* = 72)	ED− Cohort (*n* = 1082)	*p*
Micra™-AV	10 (13.8)	92 (8.5)	0.204
PM indication, *n* (%) Slow AF, *n* (%) AVB, *n* (%) Sinus node arrest, *n* (%) Cardioinhibitory syncope, *n* (%) Ablate and pace, *n* (%) Other, *n* (%)	55 (76.3) 14 (37.5) 3 (4.2) 0 (0) 0 (0) 0 (0)	538 (49.7) 296 (27.3) 178 (16.4) 32 (2.9) 21 (1.9) 5 (0.46)	**0.025** **0.027** **0.008** 0.255 0.634 1

**Abbreviations:** AF = atrial fibrillation; AVB = atrioventricular blocks; LP = leadless pacemaker; PM = pacemaker; TV = transvenous. Bold indicates statically significant values.

**Table 3:** Peri-procedural characteristics.

	ED+ Cohort (*n* = 72)	ED− Cohort (*n* = 1082)	*p*
Right femoral access, *n* (%)	69 (95.8)	1039 (96.0)	1
Femoral vein angiography, *n* (%)	16 (22.2)	358 (33.1)	0.200
Number of delivery attempts, median (IQR)	1 (1–1)	1 (1–1)	1
Patients with > 1 delivery attempt, *n* (%)	17 (23.6)	150 (13.8)	0.068
Micra™ repositioning, *n* (%)	6 (8.3)	133 (12.3)	0.453
Use of temporary pacing, *n* (%)	68 (94.4)	313 (28.9)	**<0.001**
Final positioning, *n* (%) Septum, *n (%)* RVOT, *n* (%) Apex, *n* (%)	65 (90.3) 0 (0) 7 (9.7)	718 (66.4) 24 (2.2) 340 (31.4)	0.086 0.394 **0.001**
Procedure time, mins (IQR)	60 (45–80)	50 (40–65)	**<0.001**
Fluoroscopy time, mins (IQR)	6.5 (5.0–9.7)	5.1 (3.1–9)	0.103
Overall peri-procedural major complications, *n* (%) **Pericardial effusion, *n* (%)** **Pericardial tamponade, *n* (%)** Femoral vascular injury, *n* (%) Device dislodgement or embolization, *n* (%) Groin hematoma, *n* (%)	5 (6.9) 1 (1.4) 0 (0) 1 (1.4) 0 (0) 2 (4.2)	45 (4.2) 8 (0.73) 2 (0.18) 6 (0.6) 5 (0.5) 24 (2.2)	0.244 0.443 1 0.366 1 0.676
Sensing, mV	10 (8–12.8)	10 (7.5–13.5)	0.985
Impedance, ohm	695 (570–797)	690 (580–810)	0.476
Threshold, V	0.5 (0.38–0.99)	0.42 (0.28–0.7)	0.428
Length of in-hospital stay (days), median (IQR)	7 (3–16)	3 (2–5)	**<0.001**

**Abbreviations:** RVOT = right ventricular outflow tract. Bold indicates statically significant values.

**Table 4:** Univariate and multivariate analyses for procedural times of LPM implantation.

	OR	C.I.	*p*	aOR	C.I.	*p*
Age	0.060	(−0.222–0.016)	**0.091**	0.084	(0.251–0.079)	0.308
Male sex	1.769	(−4.310–2.633)	0.636			
Coronary artery disease	2.01	(−4.812–3.094)	0.670			
CABG	3.86	(−9.768–5.419)	0.574			
Valvular HD	1.950	(−3.818–3.836)	0.997			
Hypertension	1.709	(−3.299–3.479)	0.942			
CKD treated with hemodialysis	4.007	(−5.792–9.935)	0.605			
LVEF	0.097	(0.372–0.010)	0.164			
Diabetes	2.016	(−6.118–1.796)	0.284			
Emergency LPM implantation	3.451	(6.173–19.717)	**0.000**	5.156	(4.610–24.872)	**0.004**
BMI	0.263	(0.041–0.995)	**0.072**	0.262	(0.001–1.033)	**0.049**

**Abbreviations:** BMI = body mass index; CABG = coronary artery bypass grafting; CKD = chronic kidney disease; HD = heart disease; LPM = leadless pacemaker; LVEF = left ventricular ejection fraction. Bold indicates statically significant values.

## Data Availability

The data that support the findings of this study are available from the corresponding author, upon reasonable request.
